# Supplementary material for: Causal Relationship between Aging and Anorexia Nervosa: A White-Matter-Microstructure-Mediated Mendelian Randomization Analysis
Source: Biomedicines. 2024 Aug 16;12(8):1874. doi: 10.3390/biomedicines12081874 (PMC11351342; doi:10.3390/biomedicines12081874)
Supplement: Supplementary file 1 [file biomedicines-12-01874-s001.zip › Supplementary Figure/Figure S4/Singe SNP plot of MR analysis for TL to ubm-b-1933.pdf]

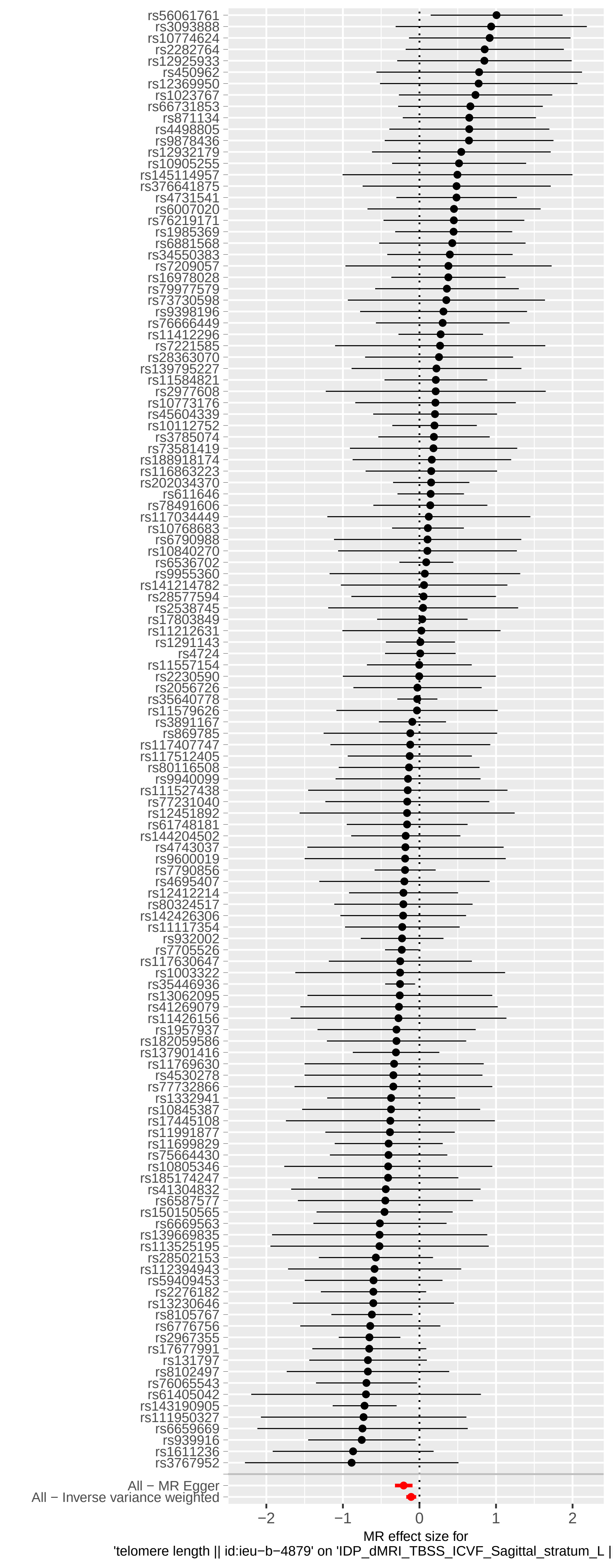
rs56061761
rs3093888
rs10774624
rs2282764
rs12925933
rs450962
rs12369950
rs1023767
rs66731853
rs871134
rs4498805
rs9878436
rs12932179
rs10905255
rs145114957
rs376641875
rs4731541
rs6007020
rs76219171
rs1985369
rs6881568
rs34550383
rs7209057
rs16978028
rs79977579
rs73730598
rs9398196
rs76666449
rs11412296
rs7221585
rs28363070
rs139795227
rs11584821
rs2977608
rs10773176
rs45604339
rs10112752
rs3785074
rs73581419
rs188918174
rs116863223
rs202034370
rs611646
rs78491606
rs117034449
rs10768683
rs6790988
rs10840270
rs6536702
rs9955360
rs141214782
rs28577594
rs2538745
rs17803849
rs11212631
rs1291143
rs4724
rs11557154
rs2230590
rs2056726
rs35640778
rs11579626
rs3891167
rs869785
rs117407747
rs117512405
rs80116508
rs9940099
rs111527438
rs77231040
rs12451892
rs61748181
rs144204502
rs4743037
rs9600019
rs7790856
rs4695407
rs12412214
rs80324517
rs142426306
rs11117354
rs932002
rs7705526
rs117630647
rs1003322
rs35446936
rs13062095
rs41269079
rs11426156
rs1957937
rs182059586
rs137901416
rs11769630
rs4530278
rs77732866
rs1332941
rs10845387
rs17445108
rs11991877
rs11699829
rs75664430
rs10805346
rs185174247
rs41304832
rs6587577
rs150150565
rs6669563
rs139669835
rs113525195
rs28502153
rs112394943
rs59409453
rs2276182
rs13230646
rs8105767
rs6776756
rs2967355
rs17677991
rs131797
rs8102497
rs76065543
rs61405042
rs143190905
rs111950327
rs6659669
rs939916
rs1611236
rs3767952
All − MR Egger
All − Inverse variance weighted
−2
−1
0
1
2
MR effect size for
'telomere length || id:ieu−b−4879' on 'IDP_dMRI_TBSS_ICVF_Sagittal_stratum_L |
